# Supplementary figures and images for: Estimating energy expenditure from wrist and thigh accelerometry in free-living adults: a doubly labelled water study
Source: Int J Obes (Lond). Author manuscript; Available in PMC 2020 Jul 13. (PMC7358076; doi:10.1038/s41366-019-0352-x)

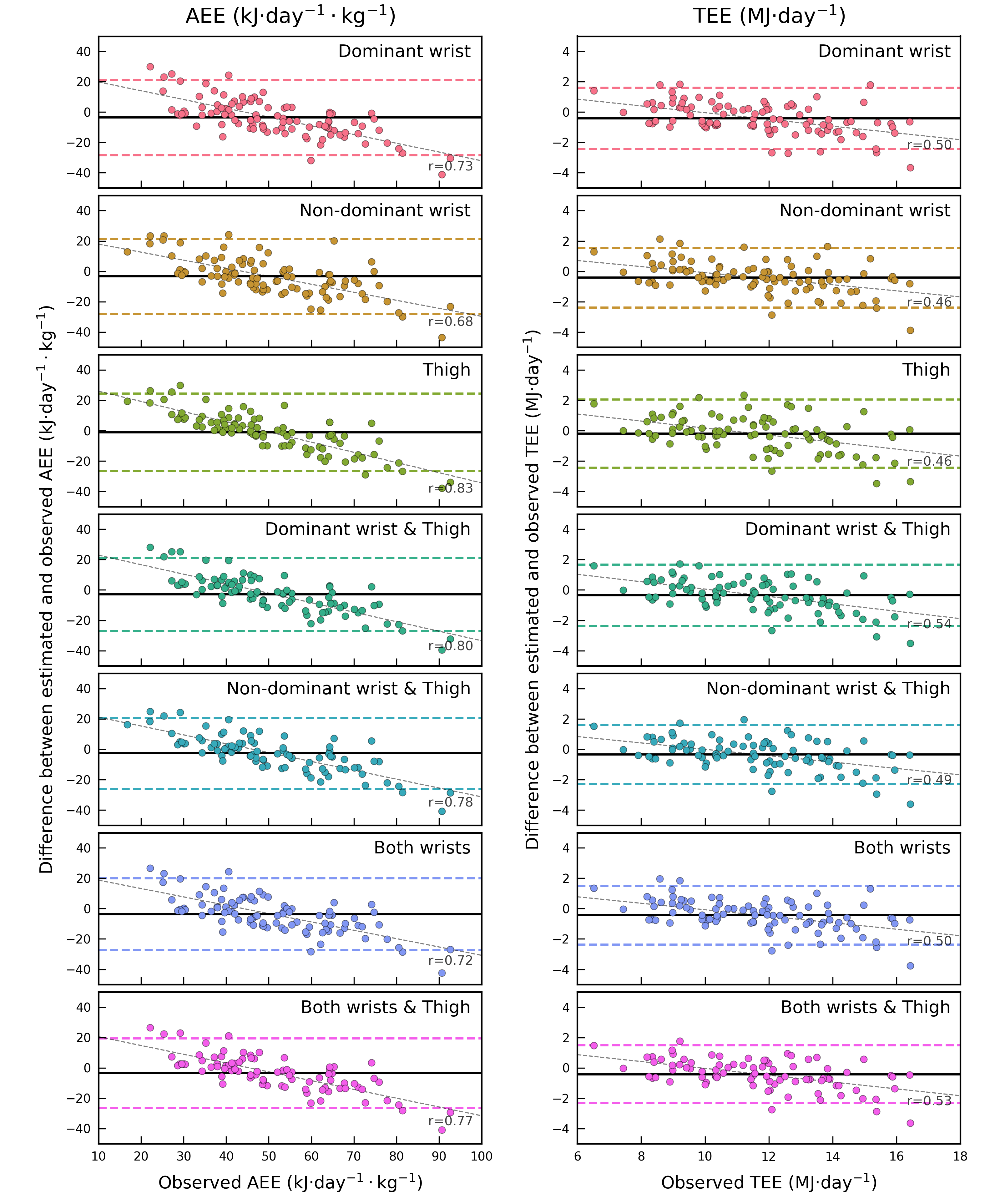

Supplement: Supplemental Figure 2 [file EMS86549-supplement-Supplemental_Figure_2.png]

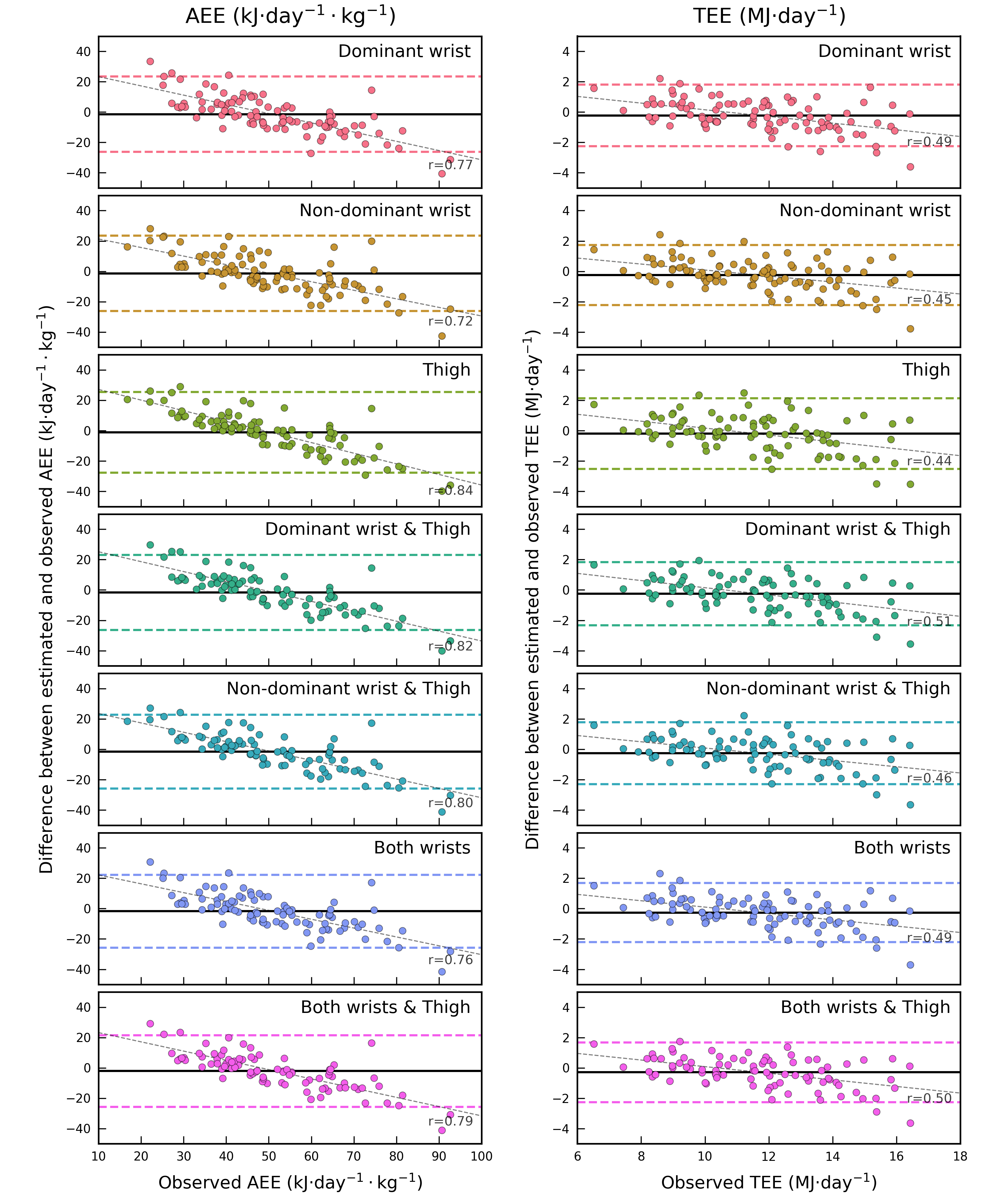

Supplement: Supplemental Figure 1 [file EMS86549-supplement-Supplemental_Figure_1.png]

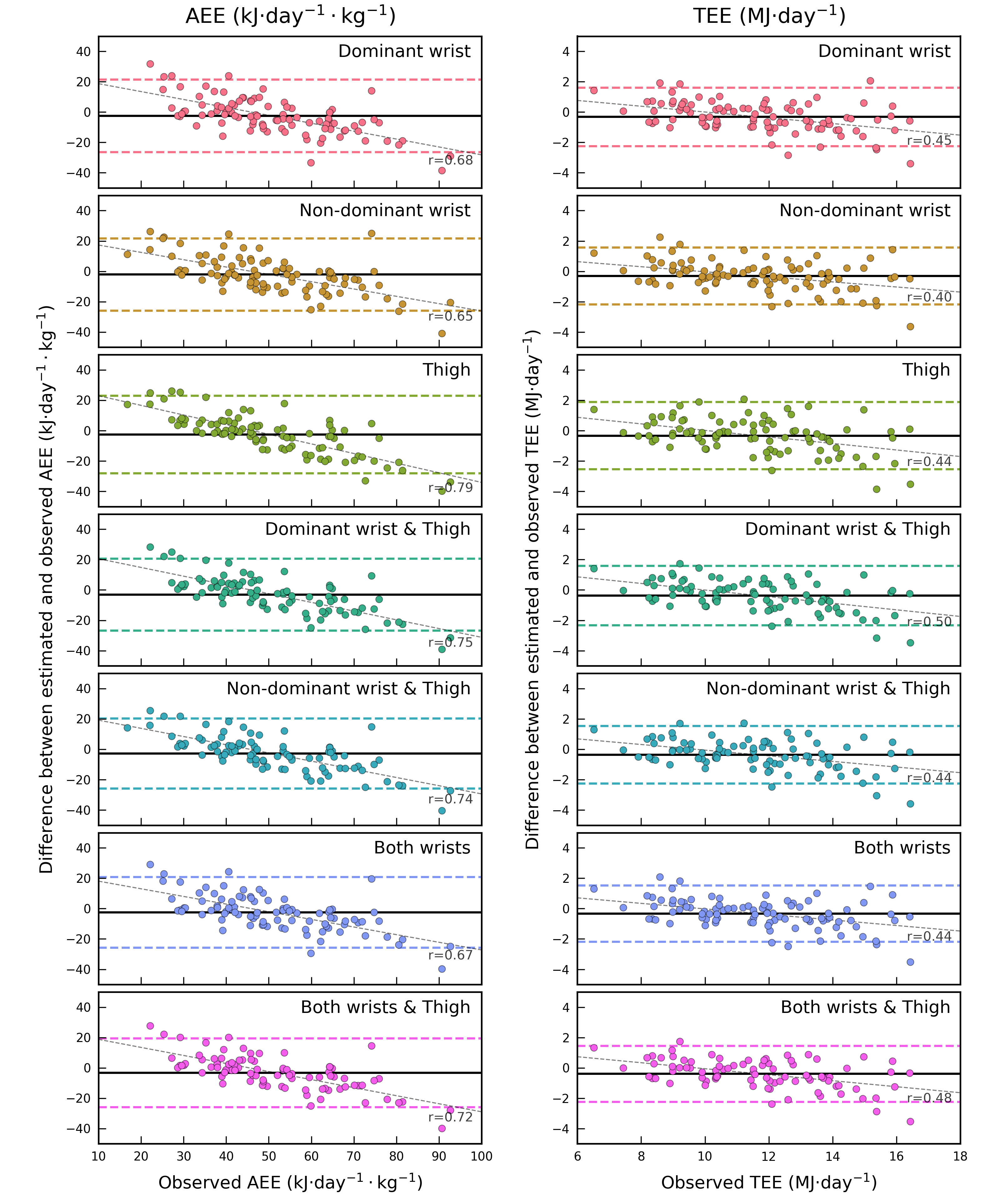

Supplement: Supplemental Figure 3 [file EMS86549-supplement-Supplemental_Figure_3.png]

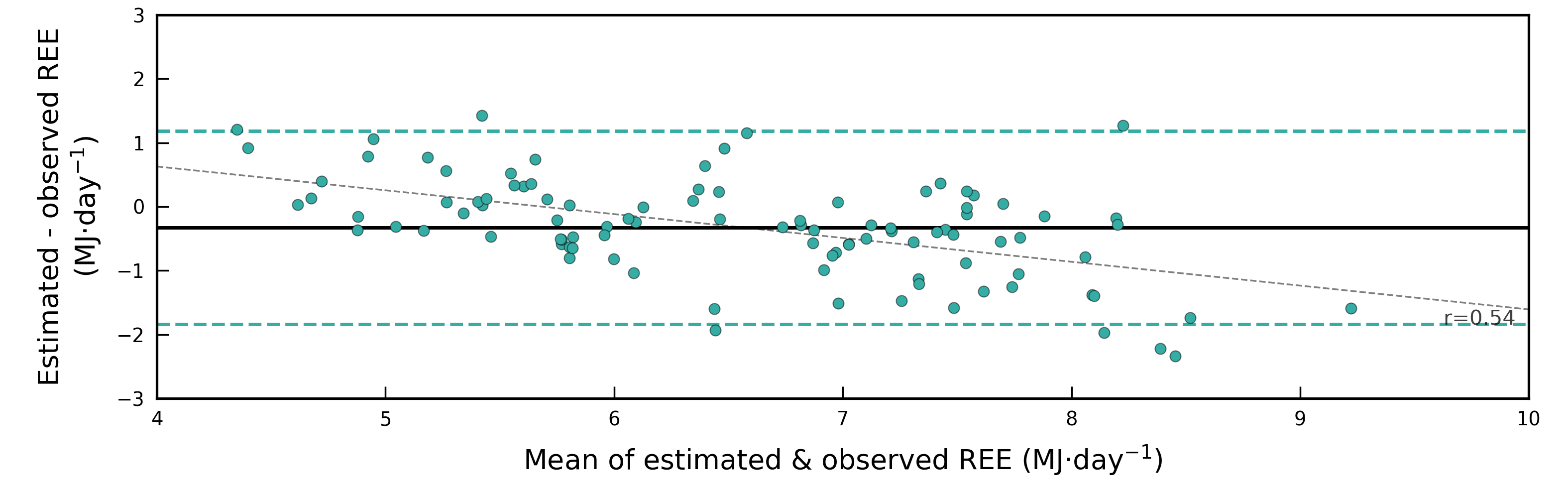

Supplement: Supplemental Figure 4 [file EMS86549-supplement-Supplemental_Figure_4.png]
