# Supplementary material for: Estimating energy expenditure from wrist and thigh accelerometry in free-living adults: a doubly labelled water study
Source: Int J Obes (Lond). Author manuscript; Available in PMC 2020 Jul 13. (PMC7358076; doi:10.1038/s41366-019-0352-x)
Supplement: Supp Data Labels [file EMS86549-supplement-Supp_Data_Labels.docx]

Supplemental Table 1: Harmonisation equations relating movement intensities between the dominant wrist, non-dominant wrist and thigh.

Supplemental Table 2: Agreement between estimated activity energy expenditure from the all models with those derived from doubly labelled water. Bias values in bold indicate statistical significance according to a paired t-test (p < 0.05).

Supplemental Table 3: Derived regression models of activity energy expenditure (normalised for body weight) using all combinations of dominant wrist, non-dominant wrist and thigh acceleration.

Supplemental Table 4: Derived regression models of activity energy expenditure (not normalised for body weight) using all combinations of dominant wrist, non-dominant wrist and thigh acceleration, including body weight.

Supplemental Table 5: Agreement between estimated activity energy expenditure from the HPFVM quadratic models with those derived from doubly labelled water, in only right-handed individuals.

Supplemental Figure 1: Bland-Altman plots illustrating agreement between the activity energy expenditure and total energy expenditure estimates from ENMO linear models with those from doubly labelled water, where the X-axis indicates the observed values.

Supplemental Figure 2: Bland-Altman plots illustrating agreement between the activity energy expenditure and total energy expenditure estimates from ENMO quadratic models with those from doubly labelled water, where the X-axis indicates the observed values.

Supplemental Figure 3: Bland-Altman plots illustrating agreement between the activity energy expenditure and total energy expenditure estimates from HPFVM linear models with those from doubly labelled water, where the X-axis indicates the observed values.

Supplemental Figure 4: Bland-Altman plot illustrating the agreement between estimated resting energy expenditure using anthropometric equations and measured resting energy expenditure during the clinic visits.
